# On Bright’s Disease

**Published:** 1853-04

**Authors:** 


### *On Bright's Disease, and its connection with Puerperal Convulsions.* By Dr. Litzmann, (Deutche Klinik.)

Professor Litzmann, of Kiel, has published an elaborate memoir on renal disorder in pregnant and puerperal females. He commences by describing twelve cases in which albuminuria and œdema were present; some of the women had convulsions. Three of the patients were pregnant with twins; and nine (including the twin cases) were primiparæ.

In several of the cases, especially where convulsions were present, or were threatened, or where there were symptoms of toxæmia, as amaurosis, etc., carbonate of ammonia was discovered in the blood and in the air expired from the lungs, and in the blood of the children who were born dead. This was in accordance with the view of Dr. Frerichs, that urea, when accumulated in the blood, becomes converted into carbonate of ammonia, and then produces toxæmic symptoms.

Including the twelve cases described, Dr. Litzmann has examined the urine of one hundred and thirty-one persons: seventy-nine during pregnancy, eighty during labor, and eighty after parturition. In these he has found albumen present in thirty-seven, and absent in ninety-five. The examinations began, with few exceptions, in the last three months of pregnancy; in almost all cases they were repeated several times, and, in several, daily; yet it is possible that small quantities of albumen may have escaped notice. In order to avoid the admixture of foreign matters from the vagina, the catheter was used, always with parturient women and those who had been delivered, and frequently in pregnant females. In the latter, as well as in those who had been confined, the urine passed in the morning was employed. Heat, and acetic, or nitric acid, were the tests used.

Of the ninety-five females, whose urine contained no albumen, fifty-three were primiparæ and forty-three multiparæ. Of the thirty-seven who had albuminuria, twenty-six were primiparæ and eleven multiparæ; among the latter was one who had albuminuria in her first pregnancy, and two who were pregnant with twins.

Among the thirty-seven females, the urine of sixteen was discovered to be albuminous during pregnancy. In ten of these the albuminuria continued during labor, and for some days afterward; in four, where it was less intense, it disappeared before confinement; and in two cases, circumstances prevented the examination from being continued.

In four women who had been confined, and whose urine contained albumen, no examination had been made during pregnancy. There can be no doubt, however, from the quantity of albumen found, and the other symptoms that it had been present.

In four females, in whose urine no albumen had been found during pregnancy, albuminuria appeared during labor — in two, evidently for the first time.

In ten persons, in whose urine no albumen had been present during pregnancy, there was considerable albuminuria after delivery. In eight of these, the urine, examined during labor, was found to contain no albumen.

In three females who had been confined, and in whose urine albumen was found, no previous examination had been made.

There is a form of albuminuria unconnected with renal disease, but arising from catarrhal irritation or blennorrhœa of the bladder. Dr. Litzmann has observed it twice during pregnancy, several times during labor, but most frequently after delivery. It generally appears on the second or third day, and goes on increasing till the sixth or seventh. The quantity of albumen is not great, and is connected with the presence of purulent mucus. At first, the urine after delivery is high colored, of acid reaction, and contains a large quantity of urates and uric acid, with epithelium and a number of pus or mucous globules. It afterward becomes lighter and yellow in color, slightly turbid, contains less urates, and is less acid; on long standing, it deposits a sediment of pus corpuscles with more or less epithelium. Fibrinous casts of the uriniferous tubes are never present. In only two cases was the quantity of pus in the urine sufficient to be detected by the naked eye: these were more chronic than most of the others, recovery not being effected till the eighth week.

In both these cases, which Dr. Litzmann relates, there had been, during labor, long continued pressure on the urethra and neck of the bladder; and this may have acted as the proximate cause of the disease. Yet cases very often occur, in which the same causes do not produce such effects. In most of the women affected with vesical catarrh after delivery, the disease could not be attributed to the above cause, the labors having been regular, and even easy. Dr. Litzmann is inclined to believe that slight vesical catarrh, which may not cause albumen to be present in the urine, but which may furnish a sufficient quantity of pus or mucous corpuscles to be detected with the microscope, is of not unfrequent occurrence after delivery. During and before labor, the disorder is much more rare. When it occurred during labor, this had always been tedious, without, however, in general, producing retention of urine. One of the pregnant females who were affected had rigors, and was at the same time seized with rheumatic swelling of the hands. In another, the vesical catarrh was brought on in the fourth month of pregnancy, by retroversion of the uterus. The local symptoms were generally very slight; on being questioned, the patients would acknowledge a little uneasiness or sense of heat in the bladder; but the state of the urine was generally the only diagnostic symptom. Of the thirty-seven cases of albuminuria which Dr. Litzmann observed, he believed that half were referrible to catarrh of the bladder; this he ascertained, in nine of the cases, by microscopic examination.

Simple albuminuria, and Bright's disease, (albuminuria accompanied by fibrinous exudation into the uriniferous tubes,) pass gradually into each other in pregnant famales. In most of the cases in which albuminuria had reached a high degree, fibrinous casts were found toward the end of pregnancy, or during labor and the early part of the subsequent period. Of the thirty-seven cases of albuminuria, Dr. Litzmann found thirteen to be connected with Bright's disease; in seven, fibrinous casts were found; in one, the urine could only be examined once, and, though not then found, they were probably present; and in five, there could be little doubt from the quantity of albumen and the concomitant symptoms—eclampsia in three cases—that they were present, although the urine was not examined microscopically.

There can be no doubt that, as has already been pointed out by Rayer, the albuminuria of pregnant females arises from a mechanical obstruction of the renal circulation. From the experiments of Frerichs, it appears that the retardation of the venous circulation on the kidneys easily produces exudation of albumen and fibrin, and finally, even of blood. On the other hand, increased arterial impulse, as from ligature of the aorta below the renal arteries, only rarely gives rise to slight albuminuria; and it is when one kidney is extirpated at the same time with the ligature of the aorta, that any notable quantity of albumen appears in the urine. In favor of the mechanical view of the explanation of albuminuria in pregnant women, may be adduced its predominance in primiparæ—a fact recognized by all observers. The tight and unyielding abdominal wall must naturally cause the uterus to press more strongly on the organs lying behind and above it. It is moreover probable, that when albuminuria has occurred in multiparæ, it has been but a repetition of the same affection which they had as primiparæ.

In many of the cases, there were also other causes which tended to increase the pressure. Five of Dr. Litzmann's patients who had albuminuria, were pregnant with twins; in others, there was a large quantity of liquor amnii, or a large child, or both; in one case, there were periodical contractions of the muscles, especially of the recti, pressing the uterus against the spinal column; in four cases, the pelvis was narrow. Three of the patients had chronic pulmonary catarrh. It cannot, however, be denied, that cases are often met with, in which all these circumstances are present without producing albuminuria; and, on the other hand, that albuminuria sometimes occurs, even in a high degree, without more than ordinary pressure being apparently exerted. An additional ground for assuming the dependence of albuminuria in pregnant women on mechanical impediment to the circulation, is found in the rapidity with which it disappears as soon as, by emptying the uterus, the free circulation of the blood has been re-established.

Besides the mechanical cause we must take into account the state of the blood in pregnant women (increase of water and fibrin, decrease of albumen, diminution of the red, and increase of the colorless particles.) Most of the pregnant women with albuminuria, observed by Dr. Litzmann, had a more or less chlorotic appearance; while others appeared fresh and healthy. The state of the blood did not appear sufficient to cause albuminuria, without an impediment to the circulation through the kidneys. In common with Lever, Devilliers and Regnault, etc., Dr. Litzmann has not been able to recognize cold or the abuse of spirituous liquors, etc., as the cause of albuminuria in pregnant females.—*London Med. Jour.*

